# SirT1 brings stemness closer to cancer and aging

**DOI:** 10.18632/aging.100272

**Published:** 2011-02-09

**Authors:** Vincenzo Calvanese, Mario F. Fraga

**Affiliations:** ^1^ Department of Immunology and Oncology, Centro Nacional Biotecnología/CSIC, Cantoblanco, Madrid E-28049, Spain; ^2^ Cancer Epigenetics Laboratory, Instituto Universitario de Oncología del Principado de Asturias (IUOPA/HUCA), Universidad de Oviedo, 33006 Oviedo, Spain

**Keywords:** Sirtuin, pluripotency, epigenetics, senescence, self-renewal, differentiation, embryonic stem cells

## Abstract

Sirtuin 1 acts in various cell processes, deacetylating both chromatin and non-histone proteins, and its role in cancer and aging has long been studied and debated. Here we discuss another aspect of SirT1 biology, its function as a stem cell pluripotency and differentiation regulator. We evaluate the implications of these findings in sirtuin inhibition-based cancer treatment and in the application of sirtuin activation for anti-aging therapy.

## Chromatin regulation in embryonic stem cells

Since its birth, epigenetics has been closely linked to development and differentiation [1]. Epigenetic processes are the main elements responsible for generating many distinct specialized cell populations from a single, undifferentiated cell. The first few cells in a zygote must be able to expand themselves (defined as self-renewal) while retaining the potential to give rise to any differentiated adult cell (defined as pluripotency for the inner cell mass and derived embryonic stem cells - ESC). To achieve this potency, germ cell epigenetic states must be reset, and the zygotic genome must be activated to allow expression of appropriate genes in subsequent development [2,3].

When a differentiation step is to be initiated, a cell must modify its gene expression program to change its phenotype. ESC are thought to have a globally open chromatin structure that keeps pluripotency maintenance genes active and differentiation genes silenced, in a reversible, highly plastic way [4]. Following differentiation towards a definite lineage, a stem cell must downregulate pluripotency genes definitively, activate expression of genes for the necessary differentiation phenotype, and lock differentiation genes for other lineages into a silent state. Moreover, these processes must be precisely regulated in space and time.

These skills are conferred on a stem cell mainly by the action of the epigenetic machinery. Specific histone marks and enzymes responsible for their establishment have been identified as central to ESC pluripotency and differentiation; these processes are based principally on the interplay of a web of key transcription factors and chromatin-modifying enzymes. Silencing of developmental genes is achieved, at least in part, through the creation of chromatin domains, termed “bivalent”, that contain overlapping regions of the transcriptionally permissive histone modification H3K4me3 and the silencing mark H3K27me3 [5]. Bivalent domains maintain silencing of developmentally important genes while simultaneously keeping them poised for either definitive repression or activation, depending on the developmental lineage for which the ESC are destined. In essence, this bivalent configuration is thought to enable retention of developmental plasticity by these genes.

Many other mechanisms are involved in the establishment of the stem cell epigenetic landscape [6]. Despite the large amount of information available on stem cell epigenetic identity, studies of histone acetylation have yet to define a clear role for this modification. Global acetylation of H3K9 increases during mESC and hESC (mouse and human) spontaneous differentiation to fibroblasts [7], whereas this residue loses acetylation during retinoic acid-induced endoderm-like differentiation of hESC [8]. The role of histone acetyltransferase (HAT) and deacetylase (HDAC) complexes in pluripotency maintenance and ESC differentiation remain only partially defined. The Sin3A-HDAC complex has a positive role in mESC pluripotency maintenance [9], whereas HDAC1 deletion in mESC leads to reduced activity of Sin3A, NuRD, and CoREST corepressor complexes; this affects only ESC differentiation, without impairing stemness or proliferation [10]. Although HAT p300 deletion does not affect self-renewal in undifferentiated mESC, it causes abnormal marker expression when differentiation is induced [9].

## SirT1, an old multi-faceted enzyme with a new role in pluripotency

Sirtuins are class III HDAC, paralogues of the yeast enzyme Sir2. They are characterized by their requirement for NAD^+^ as a cofactor, which links sirtuin activity to the cell redox state and metabolism. Sirtuin-compacted chromatin is characterized by histone lysine hypoacetylation [11]; hypoacetylation of H4K16 is the signature of Sir2 silencing [12,13]. In addition to epigenetic silencing, sirtuins have a role in various chromatin-related processes, including DNA repair, recombination and DNA replication [14,15].

Mammals have seven sirtuin family proteins, termed SirT1 to -7. SirT1, the best studied to date, has been implicated in processes as varied as metabolism, differentiation, cancer, stress response and cell senescence. Although SirT1 mutant mice generated by various targeting strategies and on different genetic backgrounds have distinct phenotypes, most of the *in vivo* data suggest that Sirt1 acts in early development [16,17,18]. This role nonetheless is unlikely to be essential, since most Sirt1 knockout (KO) mice pass through early embryonic stages, and in some cases reach adulthood; SirT1 might instead have a modulatory effect on basic developmental processes. In mESC, Sirt1 impedes p53 nuclear translocation in response to mild oxidative stress; Sirt1 downregulation allows acetylated p53 to enter the nucleus and inhibit Nanog expression, causing differentiation [19]. Nerve tissue differentiation is likewise dependent on metabolic changes and is regulated by SirT1. Under oxidative stress, mouse neural precursor cells stop proliferating and differentiate into astroglial cells (rather than neurons) through a SirT1-dependent mechanism that relies on Hes1 modulation and direct silencing of the Mash1 promoter [20]. In skeletal muscle and adipose tissue, SirT1 upregulation after fasting induces silencing of certain key genes to inhibit differentiation. In myocytes, myogenin and myosin heavy chain (MHC) genes are silenced by SirT1 through the binding and deacetylation of MyoD and the PCAF (p300/CBP associating factor) [21]. In adipocytes, SirT1 inhibits activation of genes such as fatty acid-binding protein (aP2) through recruitment of the corepressors NCoR and SMRT to the PPARgamma-response genes [22].

## SirT1 as a hESC differentiation modulator

Recent work from our lab shows that SirT1 is also strongly expressed in hESC. We demonstrated that SirT1 downregulation is necessary to establish correct, specific differentiation programs during human and mouse ESC differentiation, because SirT1 binds to and epigenetically represses a subset of developmental genes in pluripotent hESC. This enzyme exerts its repressive action on promoters, maintaining H4K16 and H4K9 deacetylated in hESC; its fine-tuned downregulation during differentiation contributes to epigenetic reactivation of these genes [23]. It is possible that SirT1 works in the context of a larger epigenetic modification complex, as demonstrated in the murine Polycomb repressive complex 4 (PRC4) [24]. Some of these SirT1-regulated genes have long been recognized as key factors in neuro-retinal morphogenesis, and their misregulation in the absence of SirT1 could account for the specific phenotype of the KO mice, which particularly affects ectodermal patterning. As predicted by the ability of the KO mice to pass through the first differentiation steps, and similar to reports for other HDAC, SirT1 does not appear to be essential in hESC pluripotency, as its depletion in these cells does not in itself induce differentiation or result in downregulation of pluripotency markers. In mouse ESC and at difference from human ESC, SirT1 depletion determines a mild negative effect on pluripotency gene expression. More importantly, Sirt1 overexpression leads to greater pluripotency gene expression, which is sustained throughout the first differentiation steps. We none-theless showed that this role of SirT1 is mostly indirect, and is probably mediated through its effect on developmental genes or through other SirT1 targets, such as p53 acetylation [17,19,23]. The importance of the precise time course of SirT1 downregulation for correct differentiation is also highlighted by the fine mechanisms that modulate its expression. In our study, we showed that SirT1 downregulation during hESC differentiation depends at least in part on CARM1. This arginine methyl-transferase helps to maintain pluripotency in mESC through direct regulation of histone methylation at the promoters of specific pluripotency genes [25,26]. We demonstrated that it also indirectly regulates differentiation genes by methylating the RNA-binding protein HuR, determining its binding and the stabilization of the SirT1 transcript. CARM1 downregulation has thus a dual role: it acts directly on pluripotency genes by regulating chromatin structure and indirectly by priming developmental genes through SirT1 downregulation. As suggested by the distinct kinetics of mRNA and protein downregulation, SirT1 decrease is probably modulated at more than one level. In mice, SirT1 protein levels are downregulated during mESC differentiation through the miR-9 upregulation that directly inhibits its translation [27]. This indicates that various mechanisms have evolved, probably with distinct relevance in different species, to ensure correct SirT1 downregulation soon after differentiation triggering.

## Linking SirT1 as a developmental gene repressor to cancer

SirT1 has long been attributed a role in cancer. The debate nonetheless remains active regarding its role as an oncogene or tumor suppressor. This controversy is based on studies of SirT1 expression in cancer and on its wide range of functions, including its activity on well-known oncogenes and tumor suppressors [28]. As a cell survival inducer, SirT1 would better fit the definition of an oncogene; for instance, p53 deacetylation and downregulation prolong survival, which could promote cancer initiation in proliferating cells through apoptosis escape [29]. SirT1 is overexpressed in many tumor types including prostate, colon, leukemia, and lymphoma [30,31,32,33]. In tumor cells, Sirt1 is located in the promoter of densely hypermethylated tumor suppressor genes (E-cadherin, MLH1, GATA-4, GATA-5 and p27) and contributes to their transcriptionally inactive state [34].

In contrast to these observations, and as SirT1 is considered important in organism survival, a tumor suppressor function might be also anticipated. In support of this theory, Sirt1 overexpression in ApcMin/+ mice induces beta-catenin deacetylation, reducing colon tumor formation [35]; in addition, both genetic and drug-induced Sirt1 activation inhibit growth and/or induce apoptosis in certain cancer models [18,30]. SuperSirt1 mice were recently generated, which have moderate Sirt1 overexpression (a ~3-fold increase); they are apparently phenotypically indistinguishable from WT mice but show metabolic differences, with increased energy expenditure and lower lipid-induced inflammation, leading to protection from hepatic steatosis [36]. Aged SuperSirt1 mice show lower DNA damage levels, are generally healthier and are partially protected from certain solid tumors [37].

SirT1-mediated modulation of stemness and developmental genes might not be merely an additional piece in the developmental epigenetic puzzle. Intense studies of sirtuins led to the development of many small molecule modulators to activate or inhibit their activity. The application of SirT1 inhibitors centers mainly on cancer therapy. Sirtinol and some of its derivatives arrest cell cycling in breast and lung cancer [38]. Both cambinol and tenovins also inhibit cancer growth and induce apoptosis [39]. Salermide treatment leads to growth arrest and a pronounced apoptotic effect in various cancer cell lines, without affecting a non-cancerous fibroblast line [40]. The discovery of a SirT1 function in suppressing developmental genes could be a further clue for the correct interpretation its role in cancer. As neoplastic cells are thought to recapitulate many stem cell characteristics [41], the oncogenic function of Sirt1 might also be mediated by aberrant regulation of developmental genes, especially for those cancers in which the undifferentiated phenotype predominates.

## SirT1 and senescence

An interesting role ascribed to Sir2 involves increased replicative lifespan, the number of cell divisions in which a mother cell can engage in its life [12]. In the worm*Caenorhabditis elegans*, in *Drosophila melanogaster* and possibly in mammals, Sir2 family members are also involved in extending lifespan [42,43]. One proposed mechanism to explain the relationship between Sir2 and lifespan is caloric restriction (CR), which increases lifespan in organisms from yeast to mammals [12]. CR produces a more oxidative metabolic state, reflected by high levels of NAD^+^. This in turn activates sirtuins and facilitates survival mechanisms such as senescence and apoptosis inhibition or activation of stress response pathways [44].

Although a mechanistic link between cell senescence and aging is lacking, the implication of SirT1 in longevity and cell senescence of most organisms suggests an important role in the aging process. A cell is considered senescent either when it reaches growth arrest owing to telomere consumption (replicative senescence) or when abnormal oncogene expression alters cell physiology (premature senescence) [45]. Data supporting a role for SirT1 in cell senescence are still correlative; senescent fibroblasts show a decrease in SirT1 expression [46] and SirT1 overexpression can inhibit oncogene-induced senescence as a result of direct p53 deacetylation, which counteracts p53 hyperacetylation in oncogene-induced senescence [47]. Many other observations support this idea, and other lines of investigation suggest an even more general role for SirT1 in aging and longevity [48].

Given the effects of SirT1 on longevity, rejuvenation and in counteracting aging, the pharmaceutical, “nutraceutical” and cosmetic industries are interested in the development of small molecule SirT1 activators [49,50]. Resveratrol, a natural compound present in traces in some red wines, has a strong sirtuin-activating effect. This polyphenol and related synthetic molecules have anti-aging activity and positive effects on aging-related disease, prolonging survival in mice [42,51,52]. Clinical trials are under way to test the therapeutic potential of sirtuin activators in conditions such as type 2 diabetes [53].

One of the more intriguing hypotheses about aging and age-related disease is that age-associated phenotypic alterations derive from the inability of resident stem cells to maintain tissue structure and function [54]. This, and our current understanding of cell senescence as summarized above, suggest that the aging process could arise from loss or misfunction of self-renewal and/or differentiation potential in adult stem cell populations. Our data show a positive role for SirT1 in stemness by aiding in the silencing of differentiation genes, which suggests new potential explanations of its ability to extend lifespan and to avoid cell and organism senescence. SirT1 might in fact contribute to maintaining a “stemness-like” status in cell populations involved in tissue regeneration and to orchestrating their correct differentiation within tissue.

**Figure 1.**
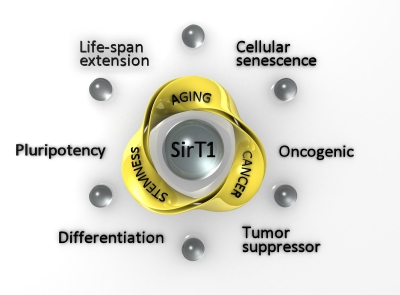
Representation of the possible connections of stemness, aging and cancer processes, mediated by SirT1. For each process, the two main effects in which SirT1 has been implicated are indicated in the external circle.

Although there is still a long path to walk before we reach full understanding of the complex and intriguing role of sirtuins, a small step has been made towards comprehension of SirT1 function in stem cells, with possible implications for the related fields of cancer and aging (Figure 1). There is hope for a solution of present controversies, for new hypotheses, and for more fruitful application of this knowledge to cancer therapy, anti-aging and regenerative medicine, to improve the quality of daily life.
